# Tunable Infrared Metamaterial Emitter for Gas Sensing Application

**DOI:** 10.3390/nano10081442

**Published:** 2020-07-24

**Authors:** Ruijia Xu, Yu-Sheng Lin

**Affiliations:** State Key Laboratory of Optoelectronic Materials and Technologies, School of Electronics and Information Technology, Sun Yat-Sen University, Guangzhou 510275, China; xurj6@mail2.sysu.edu.cn

**Keywords:** metamaterial, MEMS, IR emitter, IR sensor, mathematical fractal theory

## Abstract

We present an on-chip tunable infrared (IR) metamaterial emitter for gas sensing applications. The proposed emitter exhibits high electrical-thermal-optical efficiency, which can be realized by the integration of microelectromechanical system (MEMS) microheaters and IR metamaterials. According to the blackbody radiation law, high-efficiency IR radiation can be generated by driving a Direct Current (DC) bias voltage on a microheater. The MEMS microheater has a Peano-shaped microstructure, which exhibits great heating uniformity and high energy conversion efficiency. The implantation of a top metamaterial layer can narrow the bandwidth of the radiation spectrum from the microheater to perform wavelength-selective and narrow-band IR emission. A linear relationship between emission wavelengths and deformation ratios provides an effective approach to meet the requirement at different IR wavelengths by tailoring the suitable metamaterial pattern. The maximum radiated power of the proposed IR emitter is 85.0 µW. Furthermore, a tunable emission is achieved at a wavelength around 2.44 µm with a full-width at half-maximum of 0.38 µm, which is suitable for high-sensitivity gas sensing applications. This work provides a strategy for electro-thermal-optical devices to be used as sensors, emitters, and switches in the IR wavelength range.

## 1. Introduction

Gas sensors play a vital role in industry and also have civil applications, including as semiconductor sensors [1,2,3], catalysis sensors [4], synergistic surface reaction sensors [5], and so on. Along with the development of the Internet of Things (IOT), gas sensors with high sensitivity and selectivity have been desired for monitoring air quality in real time. Recent advances in optical gas sensors provide a path for both miniaturized and high-response speed [6,7,8]. Many studies have reported optical gas sensors using infrared (IR) absorption [9], ultraviolet (UV) absorption [10], and light scattering [11]. For gas sensing applications, IR emitters exhibit electrical-thermal characteristics by using a microelectromechanical system (MEMS) microheater, owing to its low thermal mass [12]. It utilizes conventional blackbody thermal emission in the IR wavelength range, which provides a broad-band IR emission spectrum. The thermal radiation of the microheater can be driven in a short time based on Joule heating. However, a drawback is the low thermal emission efficiency of the microheater owing to the non-uniform temperature distribution. Furthermore, the optical performance of such emitters is strongly limited by the specific operating wavelength, and it cannot be used in practical gas sensing applications.

To overcome the above-mentioned drawbacks, metamaterial is an alternative candidate for selection of a certain emission wavelength. Metamaterial has drawn a great deal of attention due to its unique optical properties, which enable it to control electromagnetic waves on subwavelength scales. The permittivity and permeability of metamaterials can be tailored by properly engineering the geometrical dimensions and material compositions of their subwavelength periodic patterns. They are widely studied to realize thermal emitters and are perfect absorbers for energy harvesting, medical imaging, and high-sensitivity sensing applications [13,14,15,16,17,18,19,20,21,22,23,24,25,26,27,28,29,30]. By tailoring the geometrical dimensions, metamaterials can be designed to span broad operating wavelengths, including visible [31,32,33], IR [34,35,36,37,38,39], terahertz [40,41,42,43,44], and microwave light [45,46]. To provide metamaterials with more flexibility, there are many techniques proposed for tuning mechanisms using MEMS technology [47,48,49,50,51,52,53,54]: liquid crystal [55], photo-excited [56], phase-change materials [57,58], thermal annealing [59], and so on. One important tuning method for metamaterial emitters is the integration of microheaters and metamaterials owing to their high efficiency. Radiation intensity can be tuned through the quantity of heating energy generated from the microheater.

In this study, we propose an on-chip tunable metamaterial IR emitter for gas sensing applications. The IR emitter is composed of a MEMS microheater and IR metamaterial. For optimization of the microheater, two structural patterns of microheaters are discussed to realize consistent high performance and heating energy conversion. The microheater is then covered with a dielectric layer to ensure electrical insulation of the device. Since the electromagnetic waves generated by the microheater have multiple polarization directions, the top metamaterial layer is tailored to be polarization independent, which can ensure uniformity of the radiation power. By properly tailoring the metamaterial patterns, it can be tuned to different wavelengths in the IR spectrum. This provides a strategy to detect various kinds of gases due to the tunable IR emitter, which can be selected at the specific absorption spectrum from the analyte. Integration of the microheater and the metamaterial achieves a tunable narrow-band emitter, which is suitable for high-sensitivity gas sensing applications.

## 2. Designs and Methods

**Figure 1:**
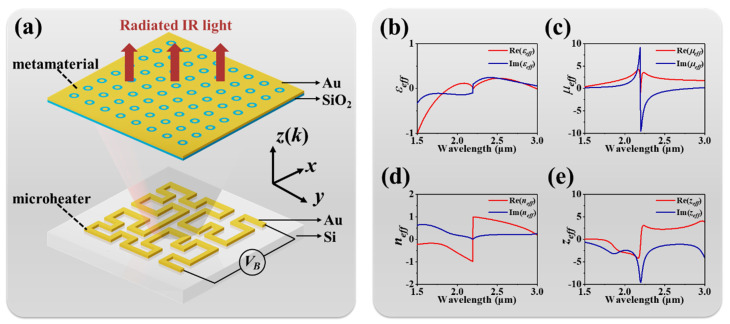
(**a**) Schematic drawing of tunable IR (infrared) emitter. The calculated (**b**) effective permittivity, (**c**) effective permeability, (**d**) effective refraction index, and (**e**) effective impedance index of the metamaterial.

Figure 1a shows the schematic drawing of the proposed on-chip tunable IR emitter. The proposed IR emitter was composed of a MEMS Peano-shaped microheater covered with a SiO_2_ dielectric layer on a Si substrate and a top metamaterial layer. Such MEMS Peano-shaped microheaters and periodic metamaterials were fabricated with gold (Au) materials at a thickness of 200-nm. The Peano-shaped microheater was covered with a SiO_2_ dielectric layer, and the metamaterial was fabricated on top to maintain electrical insulation and high energy conversion efficiency. The whole emission area of the device was 120 × 120 μm^2^. According to the conventional Joule effect, heat energy will be generated from the microheater by driving a DC bias voltage on the device. The Peano-shaped microheater was designed with a concentrated heater pattern and highly conductive metal lines, which collected all flowing electric energy and then converted it into thermal energy. The microheater achieved high electrothermal conversion efficiency and had a rapidly increasing surface temperature. Such thermal radiation power produces a broadband electromagnetic spectrum. By integrating the top metamaterial, a radiated IR light was wavelength-selected and ultimately emitted, as illustrated in Figure 1a. For the metamaterials in the proposed design, the effective permittivity and the effective permeability are expressed by the following equations [60]:

(1) $$\varepsilon_{eff}=n_{eff}/z_{eff}$$

(2) $$\mu_{eff}=n_{eff}z_{eff}$$

where *n_eff_* is the metamaterial effective refraction index, and *z_eff_* is the metamaterial effective impedance index. The effective refraction index and the effective impedance can be calculated as follows:

(3) $$n_{eff}=\frac{1}{kd}cos^{-1}\left(\frac{1-r^{2}+t^{2}}{2t}\right)$$

(4) $$z_{eff}=\sqrt{\frac{{\left(1+r\right)}^{2}-t^{2}}{{\left(1-r\right)}^{2}-t^{2}}}$$

where *r* is the reflection coefficient, *t* is the transmission coefficient, *d* is the metamaterial thickness, and *k* is the incident wavevector. The corresponding parameters of the metamaterial are shown in Figure 1b–e. While the effective refraction index was indeed positive in the resonant range, the proposed design exhibited high transmission in this range to realize a frequency-selective application. The optical properties of the designed metamaterials were studied through finite difference time domain-based (FDTD) simulations. In the numerical simulations, the incident electromagnetic wave propagated along the *z*-axis, which was perpendicular to the *x*–*y* plane. Periodic boundary conditions were defined in the *x*- and *y*-axis directions, and perfectly matched layer (PML) boundary conditions were adopted in the *z*-axis direction.

## 3. Results and Discussion

**Figure 2:**
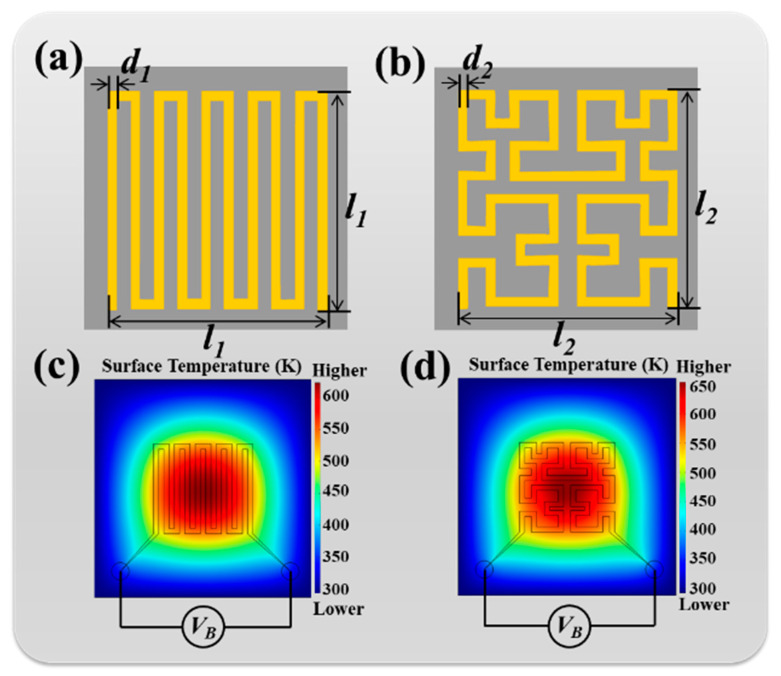
Schematic drawings of (**a**) winding microheater and (**b**) Peano-shaped microheater. (**c**,**d**) Surface temperature distributions of winding microheater and Peano-shaped microheater, respectively. Geometrical dimensions are kept as *d*_1_ = *d*_2_ = 6.0 µm and *l*_1_ = *l*_2_ = 120 µm.

Figure 2a,b shows the design of a traditional winding microheater and the proposed Peano-shaped microheater to enable comparison with the electrothermal characteristics. The pattern of the Peano-shaped microheater is a famous space-filling curve in mathematical fractal theory. For microheater design, heating uniformity is a key factor to evaluate heating performance. High heating uniformity provides high reliability of the device and prolongs its lifetime. It also results in less thermal dissipation in real-world applications, which can greatly reduce energy consumption. Herein, both microheater designs exhibited great heating uniformity, as shown in the surface temperature distributions in Figure 2c,d. By driving a DC bias voltage, most of the heating power was concentrated in the central area of the device. The effective heating area was greater than 75% (120 × 120 μm^2^), keeping a 90% maximum surface temperature, which makes such microheaters suitable for use as on-chip IR light-emitting sources with high efficiency.

**Figure 3:**
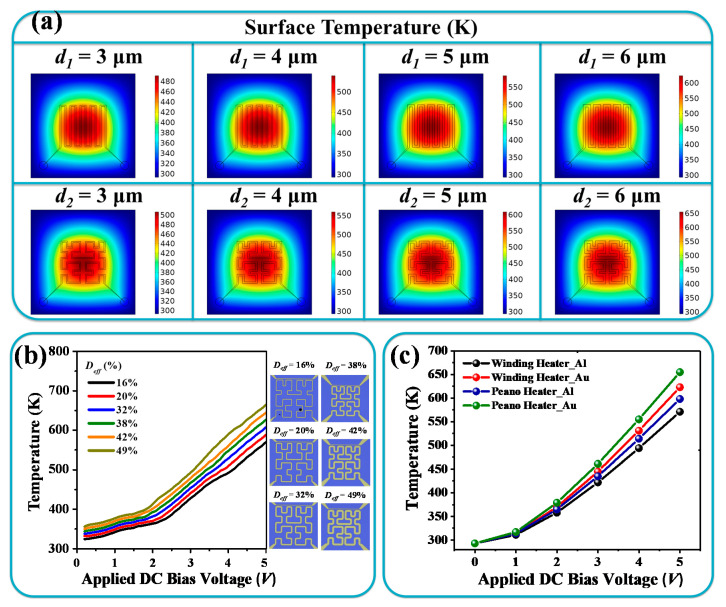
(**a**) Surface temperature distributions of microheaters with metal linewidths of *d*_1_ and *d*_2_. (**b**) Temperature as a function of driving voltage for six Peano-shaped microheater designs with different pattern density. (**c**) Relationships between applied DC bias voltage and maximum surface temperature for winding microheaters and Peano-shaped microheaters. Corresponding materials are selected as Al and Au.

Figure 3a shows the surface temperature distributions of the microheater with metal linewidths of *d*_1_ and *d*_2_. It can be clearly observed that such microheater designs exhibited great heating uniformity when the metal linewidth increased. It can also generate higher maximum temperature distribution on the surface. To further optimize the Peano-shaped microheater with high electrical-thermal efficiency, the Peano-shaped microheater was designed to facilitate comparison of six effective pattern densities (*D_eff_*, the ratio of effective heating material to the whole area of microheater) according to mathematical fractal theory. The material selected was Au, and the *D_eff_* values were 16%, 20%, 32%, 38%, 42%, and 49%, respectively. Figure 3b shows the experimental results for Au-based Peano-shaped microheaters under different driving voltages. The inserted images show top views of optical microscopes for six Peano-shaped microheaters. It can be observed that a higher *D_eff_* can realize higher surface temperature. Electrical-thermal efficiency increased along with the increments of *D_eff_*. The maximum heating temperature can reach 660 K under a driving voltage of 5 V for *D_eff_* = 49%. The fundamental electrothermal behavior is the process of energy conversion to thermal power. In the ideal situation, without considering thermal dissipation, thermal emission can be explained by Planck’s law (also known as the blackbody radiation law). According to the blackbody radiation law, emission wavelength is only related to temperature. Emission wavelength has a broad bandwidth, and emissivity is varied by the corresponding wavelength. Blackbody radiation can be expressed by

(5) $$\rho_{\lambda }d\lambda =\frac{8\pi hc}{\lambda^{5}}\frac{1}{\exp(hc/\lambda kT)-1}d\lambda$$

where *ρ_λ_* is the emission power per unit volume, *λ* is the emission wavelength, *h* is the Planck constant, *c* is the velocity of light in vacuum, *k* is the Boltzmann constant, and *T* is temperature. The heat flux flowed mainly outward on the upper and lower surfaces. Thus, half of the radiated power was ideally generated though the upper surface to be the radiation source of the proposed IR emitter. Relationship between maximum surface temperatures and applying DC bias voltages for both the winding microheater and the Peano-shaped microheater are plotted in Figure 3c. Since high-efficiency thermal conduction among metals can improve radiated power, it is also worth discussing the effect of microheaters made from different metals. The thermal conductivities of Al and Au are 238 W/(m·K) and 317 W/(m·K), respectively. By applying a bias voltage, the surface temperature increased gradually from a room temperature of 293 K. The areas of both microheater patterns were kept the same at 120 × 120 μm^2^. The maximum surface heating temperature of the Al-based winding microheater was 571 K, and it was 598 K for the Al-based Peano-shaped microheater. The maximum surface temperature of a Au-based Peano-shaped microheater can reach 660 K, while that of a Au-based winding microheater can only reach 625 K under the same driving voltage of 5 V. This shows that the radiation efficiencies of both microheaters were better when Au materials were used, as the Peano-shaped microheater generated a more complex magnetic field to induce electromagnetic heating. The maximum applied DC bias was set as 5 V to avoid breakdown of the device. Therefore, a higher surface heating temperature can be achieved by using a Au-based Peano-shaped microheater, which can generate higher radiated power. Au-based Peano-shaped microheaters are very suitable for use in tunable IR emitter design.

**Figure 4:**
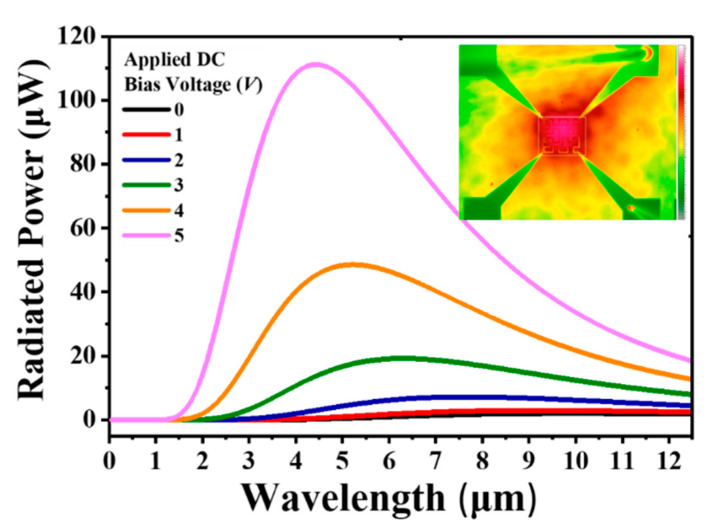
Blackbody radiated power of a Peano-shaped microheater under different applied DC bias voltage. Inset is IR thermal radiation measured with a thermal radiometer.

The relationships between the applied DC bias voltages and effective radiated powers were calculated and plotted in Figure 4. The total radiated power was below 20 µW when applying DC bias voltage from 0 to 3 V. By increasing the DC bias voltage to 4 V, the maximum radiated power was 48.6 µW. When the driving DC bias voltage was 5 V, the corresponding radiated power increased to 111.1 µW, and the emitted wavelength blue-shifted to the wavelength of 5.25 µm. The inset in Figure 4 shows the thermal radiation image measured by a thermal radiometer (Sentris thermal imaging microscope, OPTOTHERM Co. Ltd., Sewickley, PA, USA). It can be clearly observed that the temperature distribution of the Peano microheater was uniform. Moreover, the full-width at half-maximum (FWHM) of the radiated power spectrum was 5.4 µm. It is a broadband and omnidirectional IR radiation spectrum.

**Figure 5:**
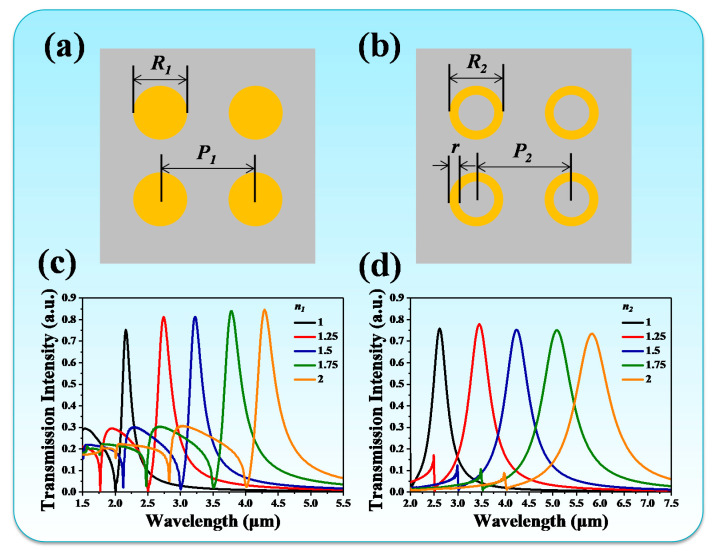
Schematic drawings of (**a**) circle-shaped and (**b**) ring-shaped metamaterials. Transmission spectra with different *n*_1_ and *n*_2_ values for (**c**) circle-shaped and (**d**) ring-shaped metamaterials. Initial geometrical dimensions were *R*_1_ = *R*_2_ = 1 µm, *P*_1_ = *P*_2_ = 2 µm, *r* = 0.2 µm.

**Figure 6:**
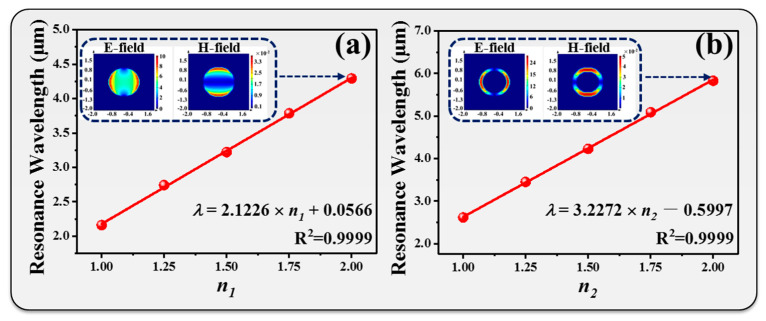
Relationships between resonances and deformation ratios for (**a**) circle-shaped and (**b**) ring-shaped metamaterials. The inserted images show the corresponding E- and H-field distributions.

To narrow down the FWHM value of the IR radiation spectrum, we proposed two metamaterial designs of Peano-shaped microheater surfaces encapsulated by insulated layers on the top. Figure 5a,b shows the proposed circle-shaped and ring-shaped metamaterials. To investigate the influence of the geometrical dimensions of the metamaterial on the emitted wavelength, the deformed metamaterial period and outer circle diameter divided by the initial metamaterial parameter is defined as *n*, i.e., the metamaterial deformation ratio. The subscripts 1 and 2 indicate circle-shaped and ring-shaped metamaterials, respectively. Figure 5c,d shows the transmission spectra of circle-shaped and ring-shaped metamaterials with different *n*_1_ and *n*_2_ values, respectively. In Figure 5c, the transmission spectrum gradually red-shifted from 2.16 µm to 4.29 µm when *n*_1_ increased from 1 to 2. The tuning range was 2.13 µm. The corresponding transmission intensity was enhanced 10% (from 75% to 85%) by increasing *n*_1_ from 1 to 2. These tuning transmission spectra are not perfect single-band resonances in the IR wavelength range. It can be clearly observed that there were several lower-order resonances at short IR wavelengths, which will result in interference with the IR emitter and will also reduce the radiated power. In order to solve this problem, a ring-shaped metamaterial was designed as shown in Figure 5d. The ring-shaped metamaterial exhibited resonance at the wavelength of 2.61 µm with a transmission intensity of 76% for *n*_2_ = 1. By increasing the *n*_2_ value to 2, the resonance red-shifted to the wavelength of 5.83 µm with a transmission intensity of 74%. The tuning range was 3.22 µm. It was enhanced 1.09 µm compared with that of the circle-shaped metamaterial. It is worth mentioning that the transmission intensity was kept stable by changing the *n*_2_ value. The variation was only 2%. The lower-order resonances were greatly reduced. The relationships between resonances and deformation ratios are summarized in Figure 6a,b for circle-shaped and ring-shaped metamaterials, respectively. To understand the physical mechanism of these tunable metamaterials, the electric (E) and magnetic (H) field distributions of circle-shaped and ring-shaped metamaterials are illustrated in the insets of Figure 6a,b, respectively. E- and H-field distributions were highly concentrated around the Au patterns and interacted with incident electromagnetic waves to provide a strong resonance. The relationships between resonances and deformation ratios were linear. This means that IR emitters can be designed at different IR wavelengths by modifying the metamaterial period. The correlation coefficients were 0.9999 for both circle-shaped and ring-shaped metamaterials. These results provide an effective approach to high-precision and wavelength-selective emission applications in the IR wavelength range.

**Figure 7:**
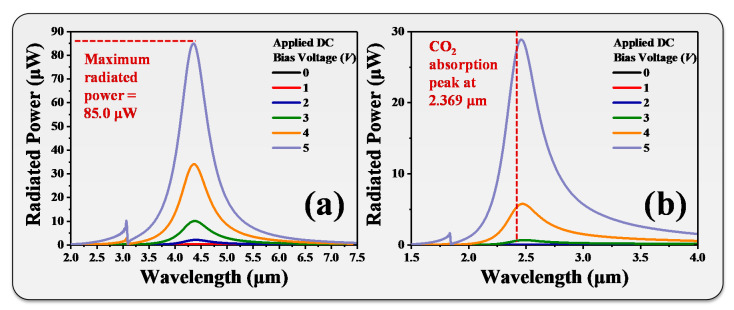
Radiated powers of the tunable emitter with (**a**) *n*_2_ = 1.542 and (**b**) *n*_2_ = 0.920 at different driving DC bias voltages for IR emitting and gas sensing applications.

According to the above results, the maximum radiated power of the Peano-shaped microheater occurred at the wavelength of 5.25 µm by driving a DC bias voltage of 5 V. We further integrated the Peano-shaped microheater with a ring-shaped metamaterial operating at a wavelength of 5.25 µm. It was promising to provide maximum radiated power with a narrow-band spectrum. The calculated deformation ratio was 1.542 (*n*_2_ = 1.542). The radiated powers of the tunable IR emitter with *n*_2_ = 1.542 at different driving DC bias voltages are shown in Figure 7a. By increasing the DC bias voltage to 5 V with a step of 1 V, the radiated powers were 0.2, 0.4, 2.1, 10.0, 33.9, and 85.0 µW for driving 1, 2, 3, 4, and 5 V, respectively. The maximum radiated power was 85.0 µW and the FWHM of the radiated spectrum was reduced to 0.65 µm. It was enhanced 8.3-fold compared to that without the ring-shaped metamaterial. Therefore, the spectrum bandwidth could be greatly narrowed and resulted in a high-efficiency, tunable IR emitter at the wavelength of 5.25 µm. Similarly, by modifying the *n*_2_ value to 0.920, the emission wavelength was 2.44 µm. The radiated powers of the tunable IR emitter with *n*_2_ = 0.920 at different driving DC bias voltages are shown in Figure 7b. Emission wavelength is matched to the absorption wavelength of CO_2_ gas at 2.369 µm. Such design is suitable for CO_2_ gas sensing applications. The FWHM of the radiated spectrum was only 0.38 µm. It was enhanced 14.2-fold compared to that without the ring-shaped metamaterial. This design opens an avenue into the potential for use in high-sensitivity gas sensing applications.

## 4. Conclusions

In conclusion, an on-chip tunable IR emitter is proposed by using a Peano-shaped microheater and IR metamaterial for gas sensing applications. By applying a DC bias voltage, high heat energy is generated via a Au-based Peano-microheater with high temperature uniformity. A surface temperature of 655 K is realized owing to the high electrothermal conversion efficiency, which generates a broadband spectrum. By properly tailoring the top metamaterial, a narrow-band IR emission is achieved. The maximum radiated power of the proposed IR emitter reached 85.0 µW. The thermally radiated emission was achieved at a wavelength of 2.44 µm, which greatly reduced the FWHM of 0.38 µm, and this matched the absorption wavelength of CO_2_ gas at 2.369 µm and can potentially be used as a high-sensitivity gas sensor. Such emitters are promising for widespread applications such as in high-efficiency IR emission and high-sensitivity gas sensing.

## References

[1] Miller D.R., Akbar S.A., Morris P.A. (2014). Nanoscale metal oxide-based heterojunctions for gas sensing: A review. Sens. Actuators B Chem..

[2] Dey A. (2018). Semiconductor metal oxide gas sensors: A review. Mater. Sci. Eng. B.

[3] Zhu L., Zeng W. (2017). Room-temperature gas sensing of ZnO-based gas sensor: A review. Sens. Actuators A Phys..

[4] Rumyantseva M., Kovalenko V., Gaskov A., Makshina E., Yuschenko V., Ivanova I., Ponzoni A., Faglia G., Comini E. (2006). Nanocomposites SnO_2_/Fe_2_O_3_: Sensor and catalytic properties. Sens. Actuators B Chem..

[5] de Lacy Costello B.P., Ewen R.J., Ratcliffe N.M., Sivanand P.S. (2003). Thick film organic vapour sensors based on binary mixtures of metal oxides. Sens. Actuators B Chem..

[6] Stanley R. (2012). Plasmonics in the mid-infrared. Nat. Photon..

[7] Kumar P., Morawska L., Martani C., Biskos G., Neophytou M.K.-A., Di Sabatino S., Bell M., Norford L., Britter R. (2015). The rise of low-cost sensing for managing air pollution in cities. Environ. Int..

[8] Hodgkinson J., Tatam R.P. (2012). Optical gas sensing: A review. Meas. Sci. Technol..

[9] Lochbaum A., Dorodnyy A., Koch U., Koepfli S.M., Volk S., Fedoryshyn Y.M., Wood V., Leuthold J. (2020). Compact mid-infrared gas sensing enabled by an all-metamaterial design. Nano Lett..

[10] Karaduman I., Yıldız D.E., Sincar M.M., Acar S., Yildiz D.E. (2014). UV light activated gas sensor for NO_2_ detection. Mater. Sci. Semicond. Process..

[11] Suematsu K., Shin Y., Hua Z., Yoshida K., Yuasa M., Kida T., Shimanoe K. (2014). Nanoparticle cluster gas sensor: Controlled clustering of SnO_2_ nanoparticles for highly sensitive toluene detection. ACS Appl. Mater. Interfaces.

[12] Rao L.L.R., Singha M.K., Subramaniam K.M., Jampana N., Asokan S. (2017). Molybdenum microheaters for MEMS-based gas sensor applications: Fabrication, electro-thermo-mechanical and response characterization. IEEE Sens. J..

[13] Ou H., Lu F., Xu Z., Lin Y.-S. (2020). Terahertz Metamaterial with multiple resonances for biosensing application. Nanomaterials.

[14] Huang W., Xu R., Lin Y.-S., Chen C.-H. (2020). Three-dimensional pyramid metamaterial with tunable broad absorption bandwidth. AIP Adv..

[15] Liang Z., Wen Y., Zhang Z., Liang Z., Xu Z., Lin Y.-S. (2019). Plasmonic metamaterial using metal-insulator-metal nanogratings for high-sensitive refraction index sensor. Results Phys..

[16] Xu R., Liu X., Lin Y.-S. (2019). Tunable ultra-narrowband terahertz perfect absorber by using metal-insulator-metal microstructures. Results Phys..

[17] Luo J., Lin Y.-S. (2019). High-efficiency of infrared absorption by using composited metamaterial nanotubes. Appl. Phys. Lett..

[18] Xu Z., Xu R., Sha J., Zhang B., Tong Y., Lin Y.-S. (2018). Infrared metamaterial absorber by using chalcogenide glass material with a cyclic ring-disk structure. OSA Contin..

[19] Xu R., Lin Y.-S. (2018). Characterizations of reconfigurable infrared metamaterial absorbers. Opt. Lett..

[20] Xu R., Luo J., Sha J., Zhong J., Xu Z., Tong Y., Lin Y.-S. (2018). Stretchable IR metamaterial with ultra-narrowband perfect absorption. Appl. Phys. Lett..

[21] Lin Y.-S., Chen W. (2018). Perfect meta-absorber by using pod-like nanostructures with ultra-broadband, omnidirectional, and polarization-independent characteristics. Sci. Rep..

[22] Landy N.I., Sajuyigbe S., Mock J.J., Smith D.R., Padilla W.J. (2008). Perfect metamaterial absorber. Phys. Rev. Lett..

[23] Watts C.M., Liu X., Padilla W.J. (2012). Metamaterial electromagnetic wave absorbers. Adv. Mater..

[24] Soukoulis C.M., Wegener M. (2011). Past achievements and future challenges in the development of three-dimensional photonic metamaterials. Nat. Photon..

[25] Chen H.-T. (2012). Interference theory of metamaterial perfect absorbers. Opt. Express.

[26] Alaee R., Farhat M., Rockstuhl C., Lederer F. (2012). A perfect absorber made of a graphene micro-ribbon metamaterial. Opt. Express.

[27] Li W., Valentine J. (2014). Metamaterial perfect absorber based hot electron photodetection. Nano Lett..

[28] Ra’Di Y., Simovski C.R., Tretyakov S.A. (2015). Thin perfect absorbers for electromagnetic waves: Theory, design, and realizations. Phys. Rev. Appl..

[29] Zhang Y., Feng Y., Zhu B., Zhao J., Jiang T. (2014). Graphene based tunable metamaterial absorber and polarization modulation in terahertz frequency. Opt. Express.

[30] Park J.-W., Van Tuong P., Rhee J.Y., Kim K.W., Jang W.H., Choi E.H., Chen L.Y., Lee Y. (2013). Multi-band metamaterial absorber based on the arrangement of donut-type resonators. Opt. Express.

[31] Lin Y.-S., Dai J., Zeng Z., Yang B.-R. (2020). Metasurface color filters using aluminum and lithium niobate configurations. Nanoscale Res. Lett..

[32] Dai J., Xu R., Lin Y.S., Chen C.H. (2020). Tunable electromagnetic characteristics of suspended nanodisk metasurface. Opt. Laser Technol..

[33] Lin Y.-S., Chen W. (2018). A large-area, wide-incident-angle, and polarization-independent plasmonic color filter for glucose sensing. Opt. Mater..

[34] Zhan F., Lin Y.S. (2020). Tunable multi-resonance using complementary circular metamaterial. Opt. Lett..

[35] Zhong J., Lin Y.-S. (2020). Electromechanically rotatable cross-shaped mid-IR metamaterial. Crystals.

[36] Wen Y., Liang Z., Lin Y.-S., Chen C.-H. (2020). Active modulation of polarization-sensitive infrared metamaterial. Opt. Commun..

[37] Mo Y., Zhong J., Lin Y.-S. (2020). Tunable chevron-shaped infrared metamaterial. Mater. Lett..

[38] Liang Z., Liu P., Lin Z., Zhang X., Zhang Z., Lin Y.-S. (2019). Electromagnetic responses of symmetrical and asymmetrical infrared ellipse-shape metamaterials. OSA Contin..

[39] Lin Y.-S. (2017). Complementary infrared metamaterials for volatile organic solutions sensing. Mater. Lett..

[40] Xu Z., Lin Z., Cheng S., Lin Y.-S. (2019). Reconfigurable and tunable terahertz wrench-shape metamaterial performing programmable characteristic. Opt. Lett..

[41] Chen X., Lin Y.-S. (2020). Polarization-sensitive metamaterials with tunable multi-resonance in the terahertz frequency range. Crystals.

[42] Xu T., Lin Y.-S. (2020). Tunable terahertz metamaterial using an electric split-ring resonator with polarization-sensitive characteristic. Appl. Sci..

[43] Yang W., Lin Y.-S. (2020). Tunable metamaterial filter for optical communication in the terahertz frequency range. Opt. Express.

[44] Zheng D., Hu X., Lin Y.-S., Chen C.-H. (2020). Tunable multi-resonance of terahertz metamaterial using split-disk resonators. AIP Adv..

[45] Shen X., Cui T.-J., Zhao J., Ma H.F., Jiang W.X., Li H. (2011). Polarization-independent wide-angle triple-band metamaterial absorber. Opt. Express.

[46] Kim Y.J., Yoo Y.J., Kim K.W., Rhee J.Y., Kim Y.H., Lee Y. (2015). Dual broadband metamaterial absorber. Opt. Express.

[47] Hu X., Zheng D., Lin Y.-S. (2020). Actively tunable terahertz metamaterial with single-band and dual-band switching characteristic. Appl. Phys. A.

[48] Ou H., Lu F., Liao Y., Zhu F., Lin Y.-S. (2020). Tunable terahertz metamaterial for high-efficiency switch application. Results Phys..

[49] Lin Z., Xu Z., Liu P., Liang Z., Lin Y.-S. (2020). Polarization-sensitive terahertz resonator using asymmetrical F-shaped metamaterial. Opt. Laser Technol..

[50] Liu P., Liang Z., Lin Z., Xu Z., Xu R., Yao D., Lin Y.-S. (2019). Actively tunable terahertz chain-link metamaterial with bidirectional polarization-dependent characteristic. Sci. Rep..

[51] Xu Z., Lin Y.-S. (2019). A stretchable terahertz parabolic-shaped metamaterial. Adv. Opt. Mater..

[52] Cheng S., Xu Z., Yao D., Zhang X., Zhang Z., Lin Y.-S. (2019). Electromagnetically induced transparency in terahertz complementary spiral-shape metamaterials. OSA Contin..

[53] Ma F., Lin Y.-S., Zhang X., Lee V.C. (2014). Tunable multiband terahertz metamaterials using a reconfigurable electric split-ring resonator array. Light Sci. Appl..

[54] Lin Y.-S., Ma F., Lee V.C. (2013). Three-dimensional movable metamaterial using electric split-ring resonators. Opt. Lett..

[55] Shrekenhamer D., Chen W.-C., Padilla W.J. (2013). Liquid crystal tunable metamaterial absorber. Phys. Rev. Lett..

[56] Zhang J., Wang G., Zhang B., He T., He Y., Shen J. (2016). Photo-excited broadband tunable terahertz metamaterial absorber. Opt. Mater..

[57] Appavoo K., Haglund R.F. (2011). Detecting nanoscale size dependence in VO_2_ phase transition using a split-ring resonator metamaterial. Nano Lett..

[58] Dao R., Kong X., Zhang H.-F., Tian X. (2019). A Tunable ultra-broadband metamaterial absorber with multilayered structure. Plasmonics.

[59] Wang B.-X., Wang G.-Z. (2017). Temperature tunable metamaterial absorber at THz frequencies. J. Mater. Sci. Mater. Electron..

[60] Smith D.R., Vier D.C., Koschny T., Soukoulis C.M. (2005). Electromagnetic parameter retrieval from inhomogeneous metamaterials. Phys. Rev. E.

